# Coexistence of Tuberculous Lymphadenitis and Hodgkin Lymphoma: A Diagnostic Challenge

**DOI:** 10.7759/cureus.82771

**Published:** 2025-04-22

**Authors:** Mridima Chandra, Maitri Parmar, Ajeet K Khilnani

**Affiliations:** 1 Otorhinolaryngology - Head and Neck Surgery, Gujarat Adani Institute of Medical Sciences, Bhuj, IND

**Keywords:** cervical lymphadenopathy, fnac, hodgkin lymphoma, ihc, tuberculosis

## Abstract

Hodgkin lymphoma (HL) and tuberculosis (TB) share overlapping clinical features, such as lymphadenopathy, fever, weight loss, and night sweats, which can complicate the diagnostic process, particularly in endemic regions. This case report describes a 10-year old boy with persistent cervical lymphadenopathy, initially diagnosed as tuberculous lymphadenitis based on fine-needle aspiration cytology (FNAC). The child was promptly started on anti-tubercular therapy (ATT), but his condition showed no improvement. Repeated biopsies ultimately revealed mixed cellularity HL, with the same lymph node testing positive for *Mycobacterium tuberculosis*, confirming a rare coexistence of both diseases. This case highlights the challenges clinicians face when both TB and HL are present, as the clinical symptoms may overlap. It also emphasizes the importance of considering malignancies as differential diagnoses in cases of persistent lymphadenopathy, even in TB-endemic regions.

## Introduction

Hodgkin lymphoma (HL) and tuberculosis (TB) are significant health challenges in TB-endemic regions where their clinical and histopathological features overlap, often leading to diagnostic dilemmas [1]. HL is a B-cell malignancy characterized by Reed-Sternberg (RS) cells in an inflammatory background, while TB presents with granulomas, necrosis, and Langhans giant cells. Both conditions share symptoms such as fever, weight loss, night sweats, and lymphadenopathy [2]. In TB-endemic areas, clinicians often lean toward TB as the initial diagnosis, particularly when granulomatous inflammation is seen on fine-needle aspiration cytology (FNAC). Diagnostic pitfalls are further exacerbated in resource-limited settings where access to molecular tools and immunohistochemistry (IHC) is restricted. However, this overlap can obscure RS cells, delaying HL diagnosis and treatment. Conversely, HL itself can induce granulomatous reactions that mimic TB histologically [3,4]. Immunohistochemistry, particularly with CD 15 and CD 30 markers, is crucial in distinguishing HL from TB, as these specifically highlight RS cells. Similarly, advanced imaging, including contrast-enhanced computed tomography (CECT) or positron emission tomography-computed tomography (PET-CT), can help delineate the disease and guide targeted biopsies [5].

The literature highlights multiple cases of HL coexisting with TB, with the diagnosis of HL only made after anti-tubercular therapy (ATT) failure or post-mortem. These cases underscore the importance of a multidisciplinary approach involving clinicians, pathologists, and radiologists. Our case highlights the need to consider HL as diagnosis in unresolved lymphadenopathy, especially when patients fail to respond to ATT. Repeat biopsies, IHC, and advanced imaging must be prioritized in such cases. Early and accurate diagnosis of HL is critical to initiating timely treatment, preventing disease progression, and improving patient outcomes.

## Case presentation

A 10-year-old boy presented to the Ear, Nose and Throat Outpatient Department of the Gujarat Adani Institute of Medical Sciences, Bhuj, Gujarat, India, with a complaint of left-sided neck swelling for two months. The patient was relatively asymptomatic two months ago and then he developed left neck swelling, which was insidious in onset, gradually progressive in nature, associated with occasional pain, fever, decreased appetite, weight loss and generalized weakness. It was not associated with cough, difficulty in swallowing, pain while swallowing, difficulty in breathing or change of voice. There were no skin lesions, abdominal pain or discomfort. He had no other co-morbidities and no other addiction history.

On inspection, multiple globular swellings were present in left neck, largest measuring approximately 5 cm x 2 cm. The margins were regular with a smooth surface, the skin over it being non-erythematous and non-edematous. The swelling did not move on deglutition or protrusion of tongue. There was no discharge, abnormal pulsations or venous prominence over the swelling. Inspection findings were confirmed on palpation. On palpation, multiple globular matted swellings were present, the largest measuring 5 cm x 2 cm x 2 cm, present over left side of neck at levels II and III. It was non-tender with normal temperature and margins merging into surrounding structures, firm in consistency, non-fluctuant, non-reducible, non-trans-illuminant, non-compressible, not fixed to underlying soft tissues and no pulsations felt over it. On percussion, a dull note was heard and on auscultation, no bruit was heard.

**Figure 1:**
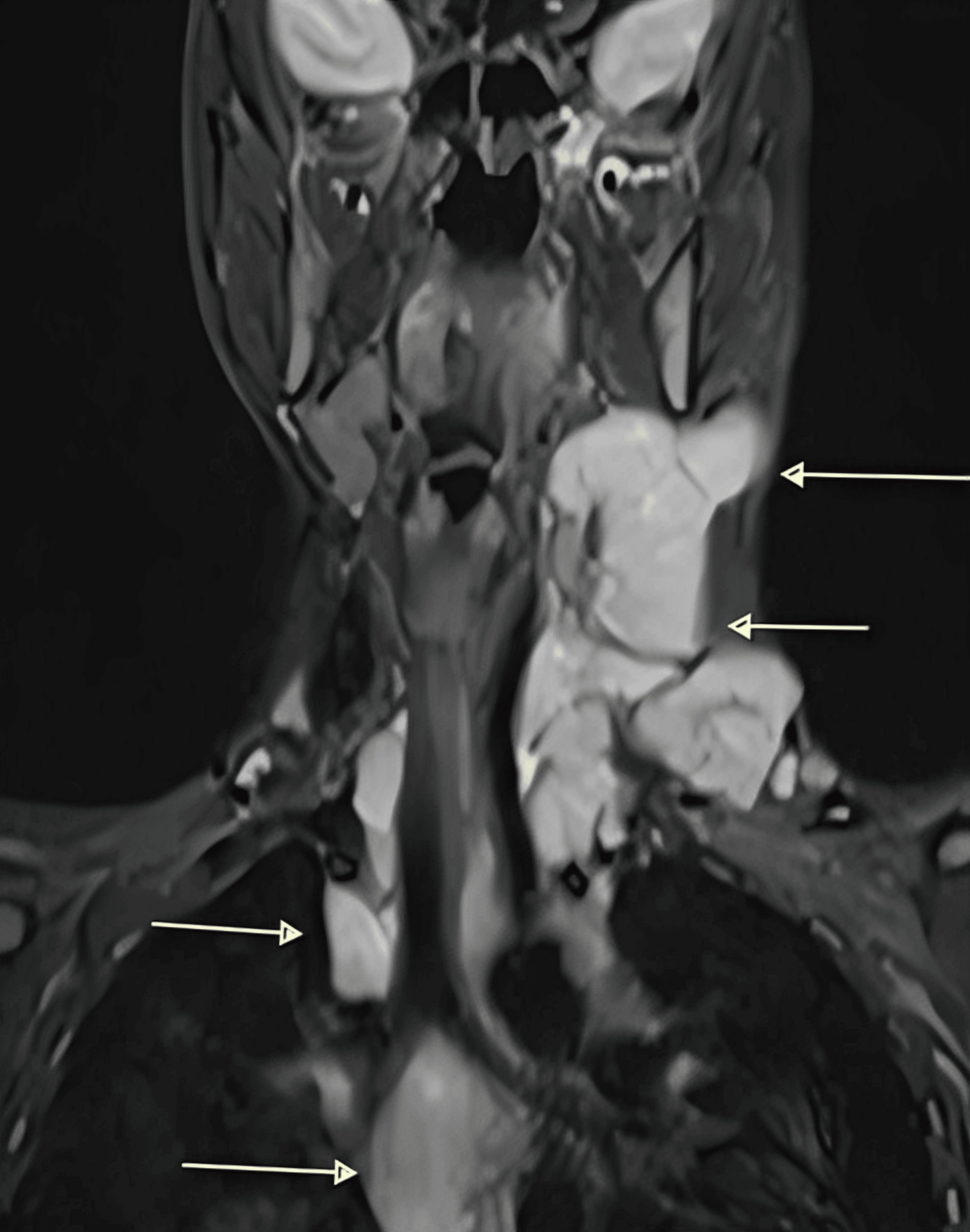
Contrast-enhanced computed tomography (CECT) of the neck showing multiple enlarged, homogeneously enhancing lymph nodes (arrow marked)

CECT of the neck was performed using submillimeter thin contiguous plain and contrast axial scan of neck with thorax, which was suggestive of multiple enlarged, homogeneously enhancing lymph nodes on left side at level II, III, IV and V stations as well as left occipital stations with mild displacement of the left internal jugular vein. Few homogenously enhancing lymph nodes were also seen on the right side. These findings raised suspicion for lymphoma (Figure 1).

**Figure 2:**
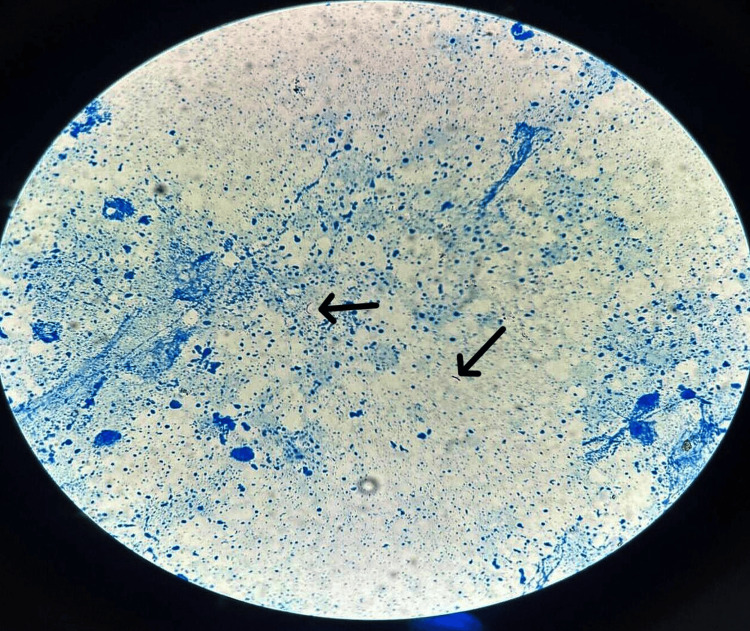
Acid-fast bacilli (AFB) (black arrow) seen in the background of necrosis (ZN stain, 1000x) ZN: Ziehl-Neelsen

FNAC, performed in June 2022, showed granulomatous lymphadenitis with well-formed epithelioid granulomas. Ziehl-Neelsen (ZN) staining was negative for acid-fast bacilli (AFB), and ATT was initiated based on clinical suspicion and cytology, as the patient’s relatives did not consent for excision biopsy at that time. A repeat FNAC (October 2022) indicated reactive lymphadenitis with a reduced node size. Excision biopsy in June 2023 showed hyalinization and fragmented tubercle bacilli, consistent with tuberculous lymphadenitis (Figure 2). 

**Figure 3:**
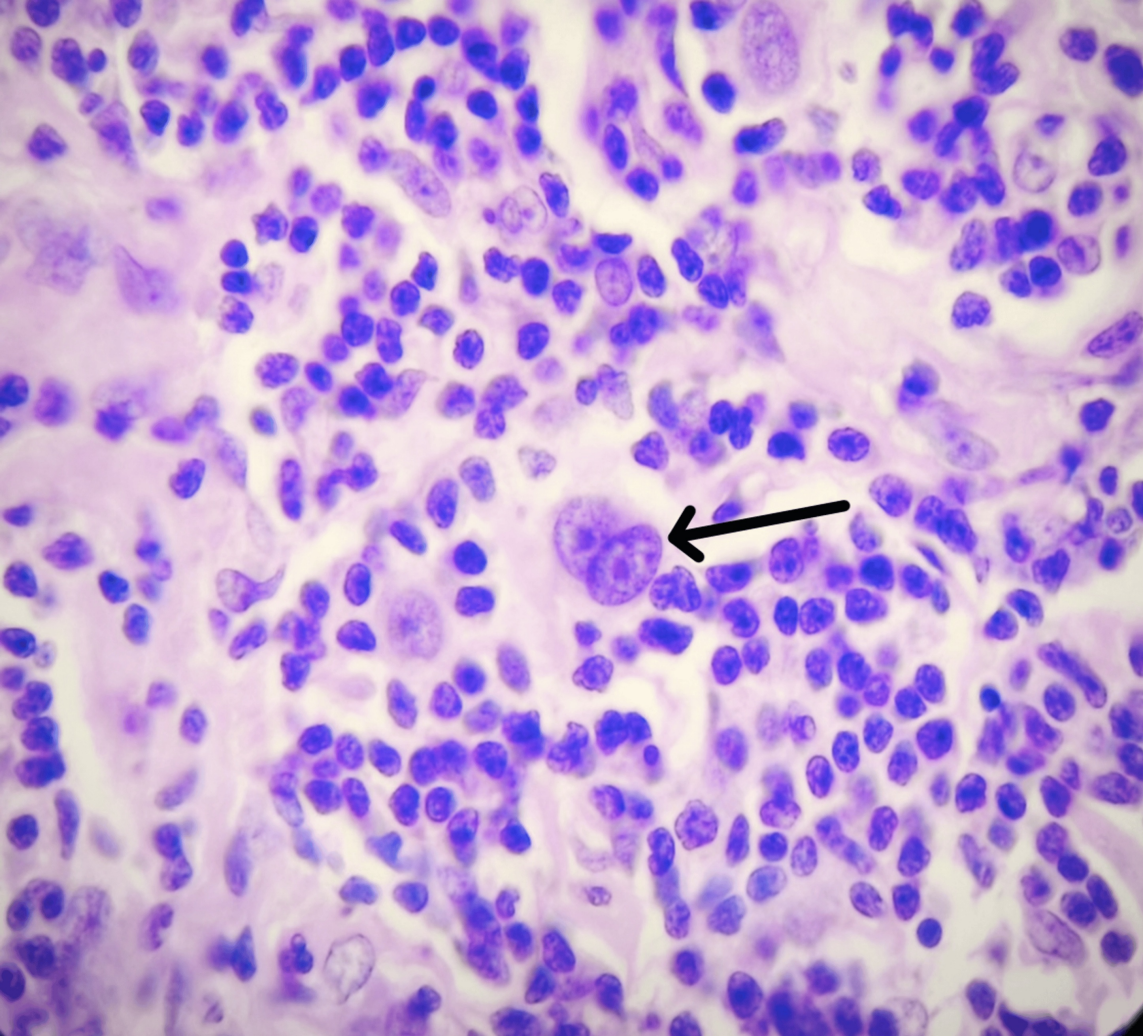
Classical RS cell (black arrow) (H&E, 400x)

The patient continued taking ATT. A third FNAC in January 2024 showed persistent lymphadenopathy with necrosis and Langhans giant cells, though ZN staining remained negative, and malignancy could not be excluded. A second biopsy in February 2024 revealed thickened nodal capsules, effaced architecture, and RS cells, confirming the diagnosis of classic HL, mixed cellularity subtype (Figure 3).

Table 1 summarizes the sequence of events leading to the diagnosis of HL.

**Table 1:** Sequence of events leading to the diagnosis of HL CECT: contrast-enhanced computed tomography; FNAC: fine-needle aspiration cytology; ATT: anti-tuberculosis treatment; CBNAAT: cartridge-based nucleic acid amplification test; MRI: magnetic resonance imaging; IHC: immunohistochemistry; HL: Hodgkin lymphoma

Date	Investigation	Report	Action taken	Time duration since initial presentation
June 29, 2022	CECT Neck	Suspicious for lymphoma		0
June 29, 2022	1st FNAC	Granulomatous lymphadenitis	ATT Started	0
June 29, 2022	Culture and sensitivity of sputum	No organism reported	-	3 days
August 11, 2022	1st CBNAAT	*Mycobacterium tuberculosis* seen	ATT continued	1.5 months
October 17, 2022	2nd FNAC	Reactive lymphadenitis	ATT Continued	3.5 months
June 15, 2023	1st excision biopsy	Tuberculous lymphadenitis with hyalinized lymphoid parenchyma (due to ATT therapy)	ATT Continued	I year
January 29, 2024	3rd FNAC	Lymphadenitis, granulomatous lesion cannot be ruled out		18 months
January 29, 2024	2nd CBNAAT	*Mycobacterium tuberculosis* not seen	ATT stopped	
February 1, 2024	CECT Neck and MRI Neck	Koch's lymphadenitis or neoplastic etiology		
February 3, 2024	2nd excision biopsy	Classic Hodgkin lymphoma, mixed cellularity subtype	Chemotherapy advised awaiting IHC report	
February 20, 2024	IHC	Positive for CD 30 and PAX-5 (Classic Hodgkin lymphoma, nodular sclerosis subtype)	Chemotherapy started	
December 20, 2024	Follow-up	Patient took six cycles of Adriamycin, Bleomycin, Vinblastine and Dacarbazine and was symptom free	Chemotherapy stopped	

## Discussion

The coexistence or diagnostic overlap between HL and TB presents a significant challenge due to their similar clinical presentations and overlapping histopathological features. Both diseases commonly present with symptoms such as fever, weight loss, and lymphadenopathy, which can easily lead to diagnostic confusion, particularly in areas where TB is endemic [3]. Additionally, granulomatous inflammation is a hallmark of TB and can sometimes be observed in HL as well, further complicating the differentiation between these two conditions. In the case discussed, the initial FNAC revealed granulomatous lymphadenitis, a finding typically suggestive of TB. This led to the initiation of ATT. However, the patient's failure to improve clinically and the persistence of lymphadenopathy raised suspicion of an alternative diagnosis, such as HL [4]. TB can mimic HL, and in such cases, misdiagnosis is not uncommon. Granulomatous inflammation and caseation necrosis, commonly seen in TB, may obscure the presence of RS cells, which are the hallmark of HL. In some instances, HL itself can induce a granulomatous reaction in response to RS cells, creating a histological appearance that resembles TB [6].

Several reports highlight the diagnostic challenges in such cases. For example, Banerjee et al. reported a similar case where HL was initially misdiagnosed as TB due to granulomatous inflammation. It was only after multiple biopsies and the patient's deterioration despite ATT that the correct diagnosis of HL was established [1]. Kunnumbrath et al. described a post-mortem diagnosis of HL in a patient who had been treated for TB, emphasizing the importance of accurate diagnosis, as delayed or missed diagnoses can have severe consequences [6]. Agarwal et al. demonstrated how IHC can be pivotal in distinguishing between HL and TB, especially in ambiguous cases. Markers such as CD 15 and CD 30 specifically identify RS cells, making them critical for confirming HL and distinguishing it from TB, where such markers are absent [7].

In resource-limited settings, where advanced diagnostic tools like IHC and molecular assays are not readily available, the challenge of differentiating HL from TB becomes even more pronounced. In such environments, repeated biopsies, advanced imaging techniques like CECT scans or PET-CT, and clinical expertise are crucial. Multidisciplinary collaboration involving clinicians, radiologists, pathologists, and oncologists is essential for making an accurate diagnosis. Clinicians should maintain a high level of suspicion for malignancies like HL in patients with persistent or unexplained lymphadenopathy, particularly if there is no clinical response to standard TB therapy. The use of advanced diagnostic techniques, even when resources are limited, can significantly improve diagnostic accuracy and patient outcomes.

The consequences of misdiagnosing HL as TB can be dire. HL is a malignancy that typically requires chemotherapy and/or radiation therapy for effective treatment. If treated as TB without considering the possibility of HL, the disease can progress unchecked, leading to worsening clinical conditions and potentially fatal outcomes. On the other hand, timely diagnosis and appropriate treatment of HL often result in favorable outcomes, especially in early-stage disease, where cure rates are high. This case highlights the importance of maintaining a high index of suspicion for HL in patients with persistent lymphadenopathy and underscores the need for early and accurate diagnosis through a combination of clinical judgment, advanced imaging, repeated biopsies, and, where possible, IHC studies [8,9].

## Conclusions

This case underscores the critical diagnostic challenge of distinguishing HL from TB in TB-endemic regions, where overlapping clinical and histopathological features often lead to treatment delays. The persistence of lymphadenopathy despite appropriate ATT should prompt clinicians to reconsider the diagnosis and pursue further investigations. As demonstrated in our patient, repeated biopsies, advanced imaging, and IHC played a pivotal role in identifying HL after an extended diagnostic journey. Early recognition and differentiation between HL and TB are vital to initiate timely oncological treatment, and ultimately improve prognosis. Clinicians must maintain a high index of suspicion and adopt a multidisciplinary approach in evaluating unresolved or atypical cases of lymphadenopathy, especially in resource-limited settings.
